# Generalization of vision pre-trained models for histopathology

**DOI:** 10.1038/s41598-023-33348-z

**Published:** 2023-04-13

**Authors:** Milad Sikaroudi, Maryam Hosseini, Ricardo Gonzalez, Shahryar Rahnamayan, H. R. Tizhoosh

**Affiliations:** 1 Kimia Lab, University of Waterloo, Waterloo, ON Canada; 2 Department of Laboratory Medicine and Pathology, Mayo Clinic, Rochester, MN USA; 3 Engineering Department, Brock University, St. Catharines, ON Canada; 4 Rhazes Lab, Department of Artificial Intelligence and Informatics, Mayo Clinic, Rochester, MN USA

**Keywords:** Computational methods, Cancer imaging, Pathology, Biomedical engineering

## Abstract

Out-of-distribution (OOD) generalization, especially for medical setups, is a key challenge in modern machine learning which has only recently received much attention. We investigate how different convolutional pre-trained models perform on OOD test data—that is data from domains that have not been seen during training—on histopathology repositories attributed to different trial sites. Different trial site repositories, pre-trained models, and image transformations are examined as specific aspects of pre-trained models. A comparison is also performed among models trained entirely from scratch (i.e., without pre-training) and models already pre-trained. The OOD performance of pre-trained models on natural images, i.e., (1) vanilla pre-trained ImageNet, (2) semi-supervised learning (SSL), and (3) semi-weakly-supervised learning (SWSL) models pre-trained on IG-1B-Targeted are examined in this study. In addition, the performance of a histopathology model (i.e., KimiaNet) trained on the most comprehensive histopathology dataset, i.e., TCGA, has also been studied. Although the performance of SSL and SWSL pre-trained models are conducive to better OOD performance in comparison to the vanilla ImageNet pre-trained model, the histopathology pre-trained model is still the best in overall. In terms of top-1 accuracy, we demonstrate that diversifying the images in the training using reasonable image transformations is effective to avoid learning shortcuts when the distribution shift is significant. In addition, XAI techniques—which aim to achieve high-quality human-understandable explanations of AI decisions—are leveraged for further investigations.

## Introduction

With artificial neural networks, model weights can be fitted to data to generate high-precision outputs, but generalization to unseen data remains challenging. These types of challenges can be addressed under different terminologies in the literature. Some works state that unsatisfactory out-of-distribution (OOD) generalization stems from learning *shortcuts*^1–3^ or *biases*^4–6^. Some other works focus on OOD generalization from a rather different perspective stating that existing *domain shift* between source and target domains is the reason behind a low OOD performance^7–9^.

We can summarize different nomenclatures describing generalization issues as follows:

- Bias
  *Definition*: Inherent or acquired prejudice or favoritism toward an entity or group of entities known as bias, or unfairness^10^.
  *Example*: Correctional Offender Management Profiling for Alternative Sanctions (COMPAS) determines whether an offender is likely to commit another crime after being sentenced. COMPAS is used by judges to decide whether to release an offender or keep him or her in prison. The software was found to be biased against African-Americans after an investigation^10^.
  *Literature*: ^4–6^.
  *Outcome*: Inducing the generalization issue on unseen data.
- Shortcuts
  *Definition*: A shortcut is a decision rule that performs well on independent and identically distributed (i.i.d.) test data but fails on OOD test data, resulting in a mismatch between what is intended and what has been learned^1^.
  *Example*: There is a tendency for cows in unexpected environments (such as beaches instead of grasslands) to be misclassified since the background can be just as significant for recognition as the cow itself^11^.
  *Literature*: ^1–3^.
  *Outcome*: Inducing the generalization issue on unseen data.
- Domain/distribution shift
  *Definition*: In the context of transfer learning any differences between the source and target domain data is known as domain shift.
  *Example*: Differences in images due to sampling bias, differences in image content or view angle, or differences in image characteristics such as brightness, noise or color^12^.
  *Literature*: ^7–9^.
  *Outcome*: Inducing the generalization issue on unseen data.

In histopathology, Hägele et al^13^ categorized 3 different types of biases in histopathology setups as below:

- Dataset bias
  For example, only a small portion of each image is correlated with its class label. For instance, a small central region of each image represents the class label and the remaining parts are irrelevant. In this scenario, the deep network cannot generalize to test images in which subjects do not necessarily lie at the center.
- Label bias
  Biases that are by chance correlated with class labels. If an image of a particular class has a unique red spot for instance, it may end up in a deep network that does not generalize to test images lacking this defect.
- Sampling bias
  The absence of certain critical tissue textures, such as necrosis, in the training, can lead to performance degradation in deep networks when testing them on not-seen textures.

Although they have coined their own nomenclature for biases, but all their types of biases are commonly known as shortcuts in the machine learning community which can result in a deep network with a low OOD while proper in-distribution performance. In addition to categorizing different types of biases in histopathology setups, they have demonstrated the effectiveness of explainable AI techniques to visualize the biases^13^.

Overall, it is critical to ensure a reliable deployment of deep models in real-life environments if there is a distribution shift, evident in differences between source and target data. For example, differences in acquisition pipelines between trial sites, or over time, may introduce a domain shift in digital pathology due to subtle and perhaps visually not apparent differences among WSIs.

A deeper understanding of distribution shift and its consequences is required to harness the significant potential offered by deep learning in histopathology. Actions need to be taken to ensure that a model’s predictions can be trusted when new data is introduced. Although correctly modeling and responding to data not seen during training is indeed a difficult problem, a few methods have recently been proposed to improve OOD generalization.

Multi-domain learning regimes (*domain generalization* and *domain adaptation*) leverage specialized training methods for OOD generalization. These types of techniques are mainly categorized into (1) simulating OOD data during training^14–16^, (2) learning invariant representations^17^, and (3) creating adversarial data acquisition scenarios^18^.

Even though domain generalization is a relatively well-studied field^19^, some works have cast doubt on the effectiveness of existing methods^20,21^. For example, Wiles et al.^22^ focused on three types of shifts in distributions, (1) spurious correlations, (2) low-data drifts, and (3) unseen shifts. Although their results were more mixed than conclusive, they suggested that simple techniques such as data augmentation and pre-training are “often" effective. They also demonstrated that domain generalization algorithms are effective for certain datasets and distribution shifts. They showed that the best approach cannot be selected a priori, and results differ over different datasets and attributes, demonstrating the need to further improve the algorithmic robustness in real-world settings. Therefore, it would be reasonable to ask whether domain generalization has progressed over a standard Expectation Risk Minimization (ERM) algorithm^22^. While those results are discouraging, there are yet other works demonstrating that machine learning models can be generalized across datasets with different distributions^22,23^. For instance, some works advocate that pre-training on large datasets is effective for OOD generalization^22,24^.

This paper presents a systematic investigation of pre-trained models for OOD generalization. Extensive experiments are conducted on different types of pre-trained models (trained with either natural images or histopathology images) with leave-one-*hospital*-out cross-validation. It means each of the WSI repositories associated with each hospital is held out in turn, and then the pre-trained models are fine-tuned using the remaining WSI repositories for the underlying task. To enable higher OOD generalization, our study focuses not on achieving state-of-the-art results on a benchmark dataset, but rather on a better understanding of how pre-trained models ensure proper OOD generalization. The results of this research should provide new insights into bridging the in-distribution and OOD gap for future research endeavors. Our contribution is three-fold:

1. In the context of OOD generalization, we show that even though pre-training on large datasets is critical (Semi-Weakly Supervised Learning (SWSL)^25^ and Semi-Supervised Learning (SSL)^25^ versus vanilla ImageNet^26^ pre-trained model), the nature of the pre-trained model is crucial as well (KimiaNet^27^ vs. SWSL^25^ and SSL^25^). A lack of one of these components may degrade OOD generalization according to our experiments.
2. With fixed-policy augmentations, OOD generalization can be improved by relying less on shortcuts and focusing more on semantically interpretable features. There is, however, a risk of complicating the deep network training as well. In other words, fixed-policy augmentation can be a friend or a foe. It all depends on the OOD test data and we may not assume a fixed-policy augmentation a priori that works for all conditions.
3. There are cases in which improving in-distribution performance may deteriorate OOD performance, showing that in-distribution performance may not be a reliable indicator of OOD performance necessarily.

In the following, we introduce different types of pre-trained models that have been investigated in this study.

### Vanilla pre-trained models using ImageNet

The pre-training paradigm is dominant in computer vision because many vision tasks are related, and it makes sense that a model trained on one dataset would help with another. As a result, the vanilla ImageNet pre-trained models, i.e., supervised learning on ImageNet1K dataset, have been dominating model training for various computer vision tasks^28–31^. Although mainly successful, some reports cast a shadow over the usefulness of vanilla ImageNet pre-trained models. For instance, Shen et al.^32^ demonstrated that vanilla ImageNet pre-training fails when we consider a much different task such as Microsoft COCO object detection^33^. Furthermore, using strong regularization, Ghiasi et al.^34^ found that a model trained with random initialization outperforms the ImageNet pre-trained model in COCO object detection. Thus, it seems one should not rely heavily on vanilla pre-trained models.

### SSL and SWSL pre-trained models

The common sense in the AI community is that a more diverse dataset for pre-training would lead to better OOD generalization. Moreover, there have been some strong pieces of evidence that pre-trained models on more diverse datasets achieve better OOD generalization in real-life distribution shifts^24,35^. For instance, there are some types of pre-trained models^25^ that have shown better performance than vanilla ImageNet pre-trained models in terms of OOD and in-distribution top-1 accuracy levels (the one with the highest probability). Among these, two promising approaches have been introduced: (1) SSL^25^ pre-trained models, i.e., pre-training on a subset of the unlabeled YFCC100M public image dataset^36^ and fine-tuned with the ImageNet1K training dataset, (2) SWSL^25^ pre-trained models, i.e., training on 940 million public images with 1.5 K hashtags and 1000 ImageNet1K synsets, followed by fine-tuning on ImageNet1K. Therefore, in this study, we investigate these types of pre-trained models to see how they perform in presence of distribution shift across different histopathology image repositories.

### KimiaNet pre-trained model

It is worth testing a deep model that has been pre-trained for histopathology. Compared to models trained with natural images, one might expect better performance from such networks. KimiaNet^27^ is a pre-trained model which has borrowed the DenseNet topology^37^ and has been trained on the most diverse, multi-organ public image repository, namely The Cancer Genome Atlas (TCGA) dataset. The details of the pre-trained models in this study has been reported in Table 1.

**Table 1:** Details of pre-trained models used in the study.

Pre-trained model	Architecture	Number of parameters	Pre-training data	Feature space dimension
Vanilla	ResNet18	11,689,512	ImageNet	512
SSL	ResNet18	11,689,512	IG-1B-Targeted, ImageNet	512
SWSL	ResNet18	11,689,512	IG-1B-Targeted, ImageNet	512
KimiaNet	DenseNet121	7,978,856	Subtying of TCGA WSIs	1024

## Experimental setup and methods

In most cases, the datasets for studying OOD performance on histopathology setups come from TCGA^16,38,39^. Given that KimiaNet^27^ has already been trained on all WSIs on TCGA data, we may not define the OOD test set from that dataset. Hence, our options are further narrowed down to other datasets. CAMELYON17 is a proper option because it contains data from various hospitals. In the following section, we describe the data and models used in our study, followed by the setup of the experiments.

### CAMELYON17 dataset

**Figure 1:**
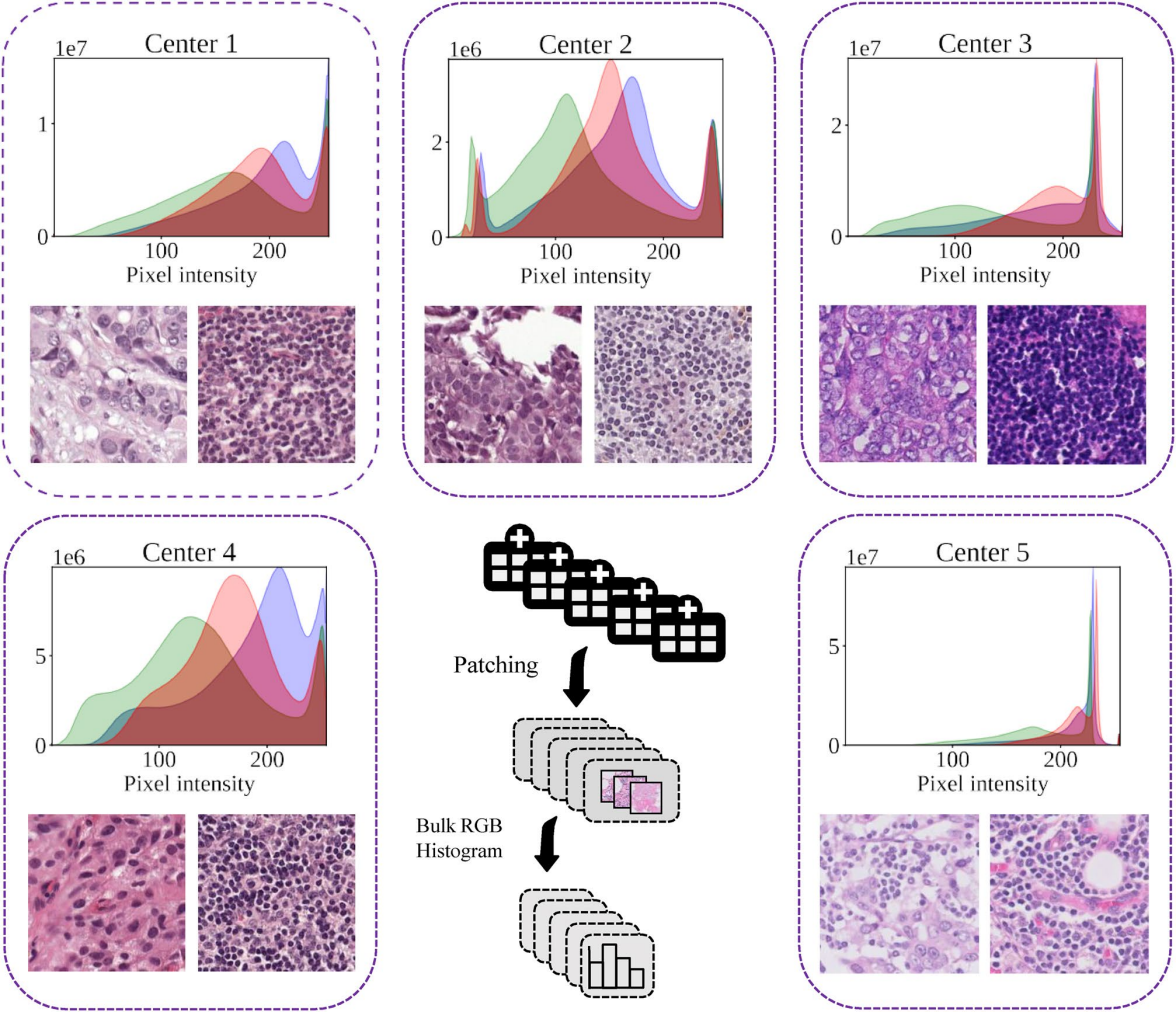
The bulk RGB histogram of the 512 × 512 extracted patches as well as sample tumor and non-tumor patches of each center/hospital in the CAMELYON17 dataset. Hospitals 3 and 5 have quite different histograms in comparison to the rest of the hospitals.

The CAMELYON17 dataset^40^ contains 1000 WSIs collected from five medical centers. These WSIs have not only spurious variations in stain colors^41^ but also variations in morphology and tumor staging across the trial sites^42,43^ (see Fig. 1). A total of 500 WSIs were used for training in the CAMELYON17 challenge, and the remaining 500 WSIs were used for testing. The training dataset of CAMELYON17 consists of 318 negative WSIs and 182 WSIs with metastases. Since only 50 WSIs of all the slides contained pixel-level annotations, only these 50 slides were sampled for tumor and non-tumor cells. Samples of non-tumor cells from the remaining slides might introduce some more variations; however, they are not likely to have any significant effect on the results^9^. Tumor areas often cover only a minor fraction of the slide area, contributing to a substantial patch-level imbalance. To address this imbalance issue, we applied a patch sampling strategy similar to that in^44^. Specifically, we sample the same number of tumor/normal patches on each slide with a uniform distribution of patches. Finally, for each hospital, we ended up with approximately 3000 patches, half of which are tumors and half non-tumors.

### OOD hospital and data chunks

In this study, for each hospital, i.e., *H*_external_, using leave-one-*hospital*-out, the backbone is only trained using the images of remaining hospitals, i.e., *H*_internal_. The *H*_internal_ is then split into training, validation, and in-distribution test set with 70, 10, and 20% chunks, respectively. The accuracy on the *H*_external_ or OOD top-1 accuracy and in-distribution top-1 accuracy are calculated during the training at each epoch.

### Different scenarios for the training data

**Figure 2:**
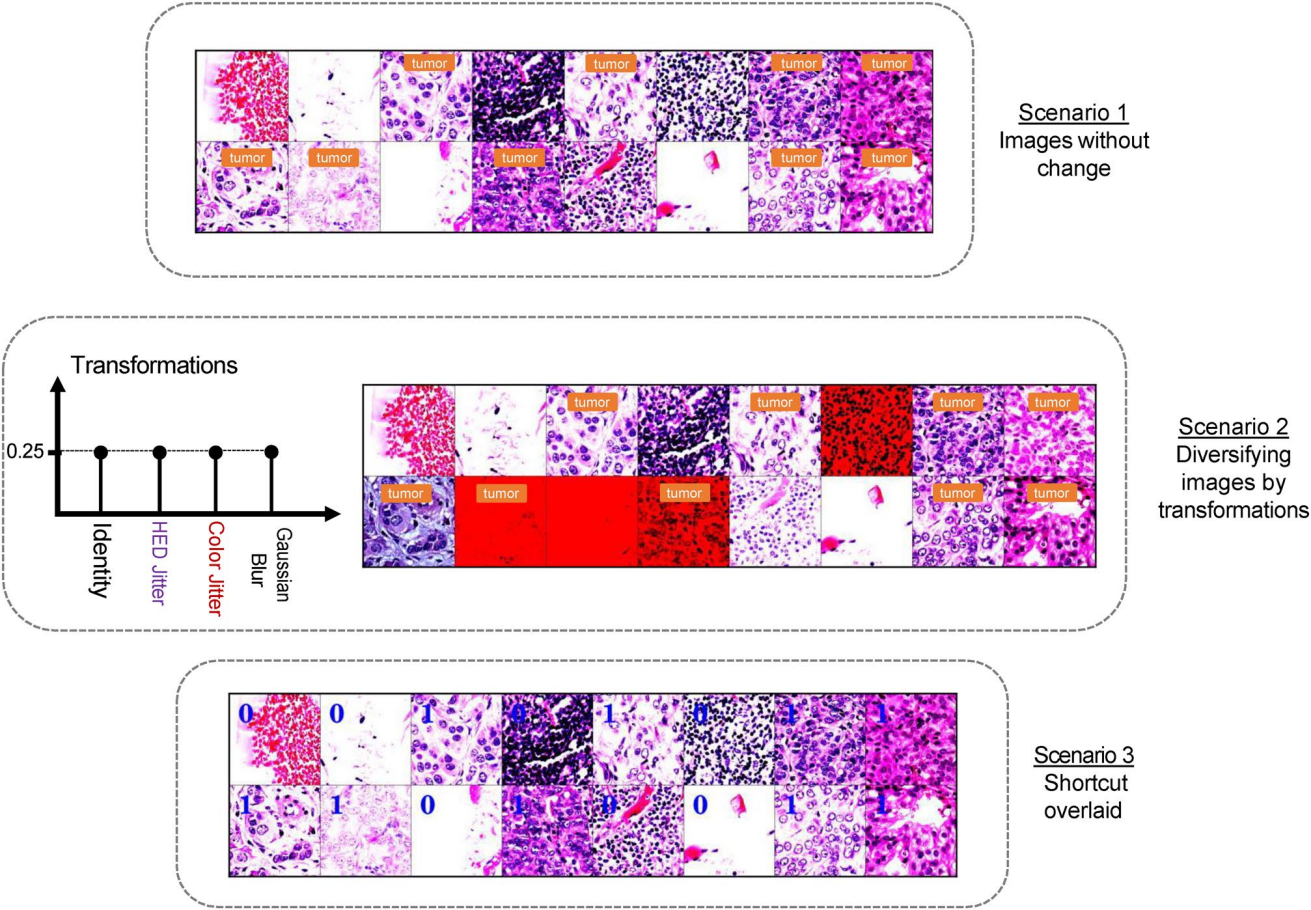
A sample training batch for different scenarios. Note that the patches in scenario 1 train sets did not undergo any augmentation. As it can be seen, among *identity*, *HED jitter*, *color jitter*, and *Gaussian blurring* transformations with uniform distribution (*p* = 0*.*25), in *scenario 2*, one transformation is picked for each image in the batch. In *scenario 3*, the correct label (0: non-tumor, 1: tumor) of each image is overlaid on the image itself.

Here, we propose different scenarios for fine-tuning or training the models in our experiments. To this aim, three different scenarios for the training are assessed according to Fig. 2 as follows:

*Scenario 1*: The training images, i.e., *H*_internal_, are fed to the network without any changes.

*Scenario 2*: Several types of distortions in histopathology setups (see Fig. 2) are simulated and randomly (uniformly) applied to *H*_internal_, before inputting these images to the deep network. These transformations are as follows:

- HED jitter^45^ randomly perturbs the HED color space value on an RGB histopathology image. Firstly, the hematoxylin and eosin color channels are separated by a color deconvolution method^46^. Following that, the hematoxylin, eosin, and Diaminobenzidine (DAB) stains are perturbed independently. In the end, the resulting stains are transformed into regular RGB color space. These perturbations are expected to make the model stain invariant.
- Color jitter directly perturbs the image attributes including brightness, contrast, and saturation to increase image diversity so that the model is more robust to variations in color.
- Gaussian blurring adds some blurring using a Gaussian kernel with a specific radius.
- Identity does not make any changes to the input images.

*Scenario 3*: A digit (0: non-tumor and 1: tumor) corresponding to the label of images are overlaid on the top left corner of each image according to Fig. 2. We kept aside this experiment for the later sections when the shortcut learning is discussed.

### Training the pre-trained models

The vanilla ImageNet pre-trained model as well as SSL^25^ and SWSL^25^ pre-trained models, all on the ResNet18 backbone^47^, have been used and assessed. In addition to pre-trained models on natural images, the KimiaNet pre-trained model^27^, which is a domain-specific (histopathology) model, has also been assessed.

For all the experiments, according to^48,49^, the total batch size was 32, and base learning rate was set to 0.01 for the *training-from-scratch* cases, and 0.001 for the pre-training cases along with step-LR schedule of 7 steps and *γ* = 0*.*1. Stochastic Gradient Descent (SGD) optimizer, a frequently used optimizer in the literature^50^, was used for the training with the weight decay value of 1*e* − 4.

## Results and discussion

During the experiments, it was observed that the in-distribution test accuracy was increasing almost smoothly during the training, while the OOD top-1 accuracy did not follow the same pattern. The underlying reason behind oscillating OOD top-1 accuracy across the epochs is that most likely during the training, a combination of both semantic and non-semantic features are learned. The non-semantic (or hospital-specific) features would counter-intuitively degrade the generalization of the OOD test data. In what follows, we compare different types of pre-trained models in terms of OOD performance.

### OOD performance of the pre-trained models

#### From scratch versus pre-trained

The first observation was that pre-trained models outperform *training from scratch* on average by far. According to Table 2, the difference between *training from scratch* and pre-training is significant. One might conclude that using any types of reasonably pre-trained model is better than *training from scratch* when it comes to OOD generalization. This finding has already been reported in^49^.

**Table 2:** The OOD performance of *training from scratch* versus the pre-trained models (vanilla, SSL, and SWSL).

Pre-training	Weights	Training scenario	Hospital 1	Hospital 2	Hospital 3	Hospital 4	Hospital 5	Average
F	Random	S_1_	93.01	89.22	84.95	91.06	81.09	87.9 ± 4.22
F	Random	S_2_	92.72	90.28	82.01	90	80.71	87.1 ± 4.73
T	Vanilla	S_1_	98.75	96.03	94.42	96.65	90.54	95.3 ± 2.69
T	Vanilla	S_2_	98.62	93.6	97.06	97.19	91.67	95.6 ± 2.52
T	SSL	S_1_	98.52	96.92	94.8	97.46	96.61	96.9 ± 1.19
T	SSL	S_1_	99.18	94.98	95.09	97.79	97.21	96.8 ± 1.59
T	SWSL	S_2_	99.08	96.52	94.97	98.12	83.93	94.5 ± 5.37
T	SWSL	S_2_	99.31	96.19	97.44	98.09	89.71	96.1 ± 3.3
		Average	97.4 ± 1.95	94.2 ± 2.05	92.6 ± 4.01	95.8 ± 2.29	88.9 ± 4.48	

Each column represents the OOD top-1 accuracy on the hold-out set.

For the *training-from-scratch* cases, according to Table 2, *scenario 2* underperforms *scenario 1* in most cases. For the pre-trained models, according to Table 2, *scenario 2* outperformed *scenario 1* when the hold-out dataset was hospitals 2, 3, or 5. In other words, by starting from proper initial weights (pre-trained), adding complications (augmentation/diversification) to training would result in a more generalized model. In contrast, if the deep network does not start with a proper initial weight (*training from scratch*), adding complexity to training would confuse it and cause it to deviate from learning meaningful and semantic features.

#### Vanilla versus SSL and SWSL

In Table 2, the maximum performance on each hold-out hospital has been highlighted. Neither *training from scratch* nor the vanilla pre-trained model has been highlighted in none of the cases. It can be observed that SSL^25^ and SWSL^25^ pre-trained models are decent alternatives for the vanilla pre-trained model. This can be justified since these two pre-trained models, i.e., SSL^25^ and SWSL^25^, have been pre-trained on a larger and more representative dataset enabling them to preserve more generic features. There have been some experiments in which training with *scenario 2* has degraded OOD performance, for example, when hospital 2 was the hold-out set. Considering that this hospital has a smaller number of images than other hospitals (≈ 2000 to ≈ 3000), it may be suggested that image diversification/augmentation lowers performance as it increases the risk of complicating training of the deep network.

#### KimiaNet

Table 3 summarizes the result of the KimiaNet for (1) linear probing^51^ (which freezes the feature extractor and trains only the classification head), and for (2) fine-tuning (all the model parameters are updated). As apparent from Table 3, the results of the fine-tuning outperformed linear probing. The average results in hospitals 2, 3, and 5 are lower and more variable in comparison to hospitals 1 and 4. Training using *scenario 2* has outperformed *scenario 1* when the hold-out trial site was hospitals 1, 3, and 5.

**Table 3:** The OOD performance of linear-probing versus the fine-tuning of KimiaNet.

Fine-tuning v.s. Linear-probing	Training scenario	Hospital 1	Hospital 2	Hospital 3	Hospital 4	Hospital 5	Average
Fine-tuning	*S*_1_	98.75	96.6	96.44	98.58	95.54	97.2 ± 1.24
Fine-tuning	*S*_2_	99.18	95.95	99.18	98.45	97.85	98.1 ± 1.17
Linear-probing	*S*_1_	97.59	86.77	92.45	95.58	80.57	91.2 ± 5.82
Linear-probing	*S*_2_	96.77	86.77	94.71	96.7	91.62	93.3 ± 3.69
Average	98*.*1 ± 1*.*08	92*.*3 ± 4*.*69	95*.*7 ± 2*.*78	97*.*3 ± 1*.*42	91*.*4 ± 7*.*51		

Each column represents the OOD top-1 accuracy on the hold-out (external) hospital.

Considering both Tables 2 and 3, KimiaNet outperformed all the other pre-trained models at least for three of five external validations. Hence, the domain-specific (histopathology) pre-trained model is conducive to better OOD generalization. Although linear probing, in both *scenario 1* and *scenario 2* cases, has outperformed *training from scratch*, it has underperformed all the fine-tuning cases regardless of the utilized pre-trained model.

#### Hospitals 3 and 5 OOD versus in-distribution performance

**Figure 3:**
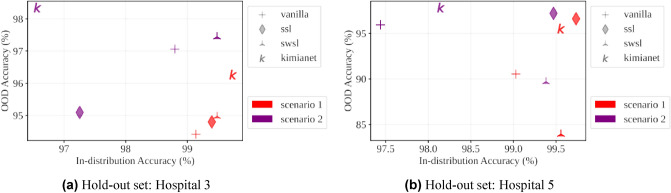
The OOD versus in-distribution top-1 accuracy for the model trained using *scenario 1* versus *scenario 2* for the hospitals 3 and 5 with significant distribution shift relative to other hospitals.

The variation in accuracy and performance in Tables 2 and 3 between pre-trained models and *training-from-scratch* for hold-out hospitals from *training-from-scratch* shows that deep networks perform the least among the other holdout hospitals when hospitals 2, 3 and 5 are the hold-out hospitals. Hospital 2 in comparison to the other hospitals has a lower amount of patches (≈ 2000 vs. ≈ 3000) so the variability of results and lower performance when it is used as the hold-out OOD hospital is justifiable. However, there is a need to investigate hospitals 3 and 5 since their variability can indicate that these two medical centers are likely to have a disproportionate distribution shift from others. The OOD versus in-distribution accuracies has been plotted according to Fig. 3.

Better OOD performance is preferred when it comes to real-world applications. Among different pre-trained models, we can see that when the hold-out set was hospital 5, KimiaNet outperformed other pre-trained models in terms of OOD performance. SWSL^25^, vanilla, and SSL^25^ pre-trained models secured the next places when training using *scenario 2* is considered. However, in *scenario 1* KimiaNet secured the first rank, and SWSL, SSL, and vanilla pre-trained models came after respectively.

Another observation was that training using *scenario 2* improves OOD performance better than *scenario 1* while worsen in-distribution performance for all types of pre-trained models. In other words, the transformations used in *scenario 2* are effective in improving OOD performance while they caused degrading the in-distribution performance. This implies that in-distribution accuracy cannot be an indicator of OOD performance necessarily. For instance, the KimiaNet in *scenario 2* has the worst in-distribution performance while the best OOD performance. It is also a noteworthy point that KimiaNet in *scenario 1*, when the hold-out hospital was hospital 3, has the best in-distribution and OOD performance while *scenario 2* boosted the OOD performance at the cost of degrading in-distribution performance. This is the case for *shortcut learning* since the shortcuts, based on our general understanding, make satisfactory in-distribution performance while degrading OOD performance. Hence, we further study this case using XAI techniques to shed light on possible shortcuts.

### Uncovering *shortcut* learning

Neural networks (or any machine learning algorithm) generally implement decision rules that define a relationship between input and output, e.g., assigning a category to each input image in classification tasks. Relying on *shortcuts*, the network performs well on training and in-distribution tests but not on OOD tests, indicating a mismatch between intended and learned solutions^1^.

#### Shortcut v.s. bias

In machine learning, bias is any kind of favoritism toward an entity^52^. Favoritism can be directed toward a specific race, or it can be directed toward particular data from a specific hospital, or even some data with specific characteristics. These types of favoritism may/may not lead to shortcut learning. It may be assumed that a bias exists when just the images from a single specific trial site are included in the training of the deep network. As a result, we would train a biased deep network that could/could not perform decently on OOD test images. The diversity of the images in that trial site determines the outcome. We may encounter a generalization issue if the images from a particular trial site are not diverse enough. Using *scenario 3*, we simulate a scenario in which the training images contain meaningful digits indicating their true labels. We may bias our results in favor of images with overlaid labels in this manner. While this bias results in satisfactory results when tested on images with overlaid labels, it causes the deep network to ignore the remaining contexts of the images, i.e., become biased towards the overlaid labels. In overall, all shortcuts can be termed as a bias but not all biases can be assumed as shortcuts necessarily. In other words, among all types of biases, those that end up with high in-distribution performance and low OOD performance are referred to as biases.

For all the experiments in this section, we used KimiaNet^27^ with the same hyperparameters that we already used in former sections, with hospital 3 as the hold-out set.

#### Scenario 3

As experimental shortcuts, one can overlay the true labels on the training images. When the deep network is trained using *scenario 3*, it may use this opportunity during training, and most likely some decision rules are learned based on this shortcut opportunity. This type of shortcut are termed as *label bias* in the literature^13^. The network will not take into account the intended and general features of the tissue context but rather the overlaid label digit. When an image without an overlaid label is tested after training, since the network has not learned meaningful decision rules, it will just output the overlaid category.

**Figure 4:**
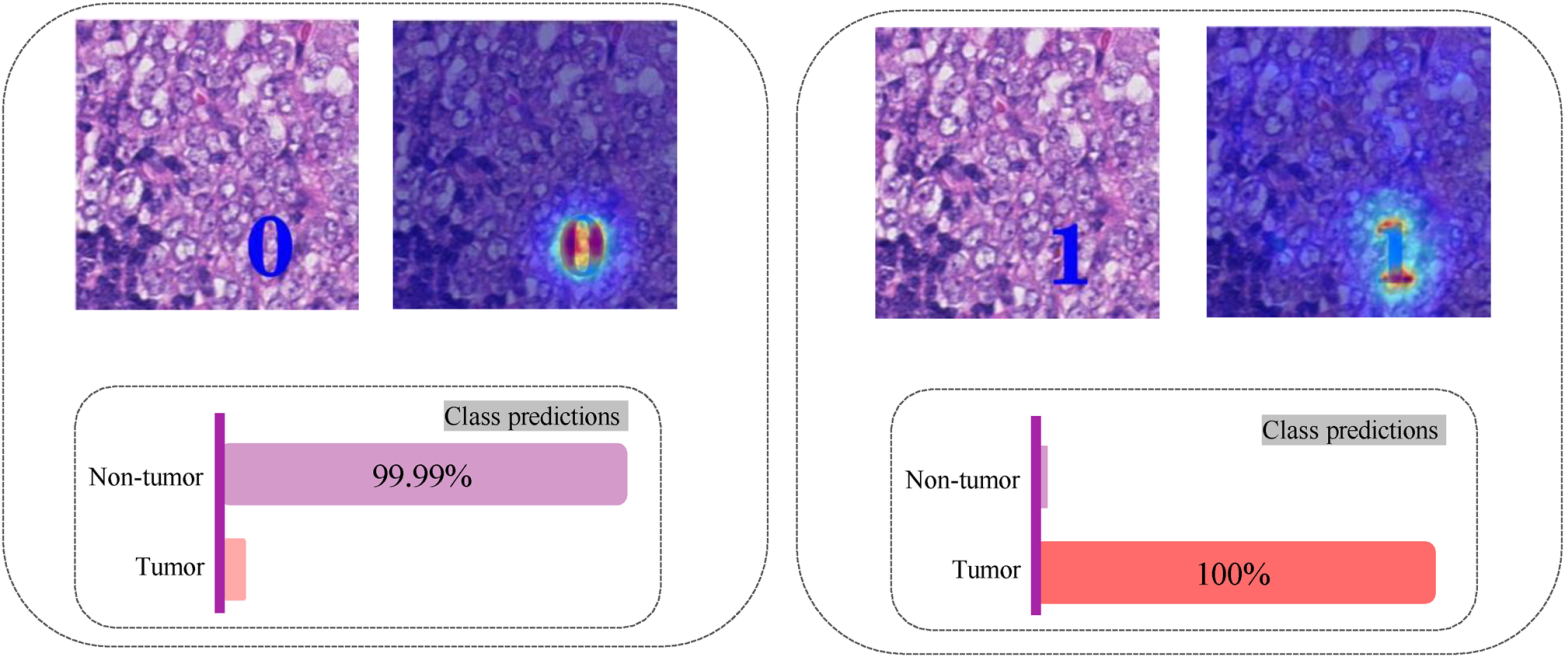
KimiaNet trained using *Scenario 3* when tested with a tumorous OOD patch with different class labels overlaid and their corresponding GradCAM heatmaps. (left) When false label (0: non-tumor) has been overlaid on the image. According to the class prediction of the network, the network has thoroughly paid attention to the overlaid digit and misclassified the image with its misleading shortcut. (right) When the true label (1: tumor) has been overlaid. The network, by focusing on the shortcut, classified the patch with a high degree of certitude.

**Figure 5:**
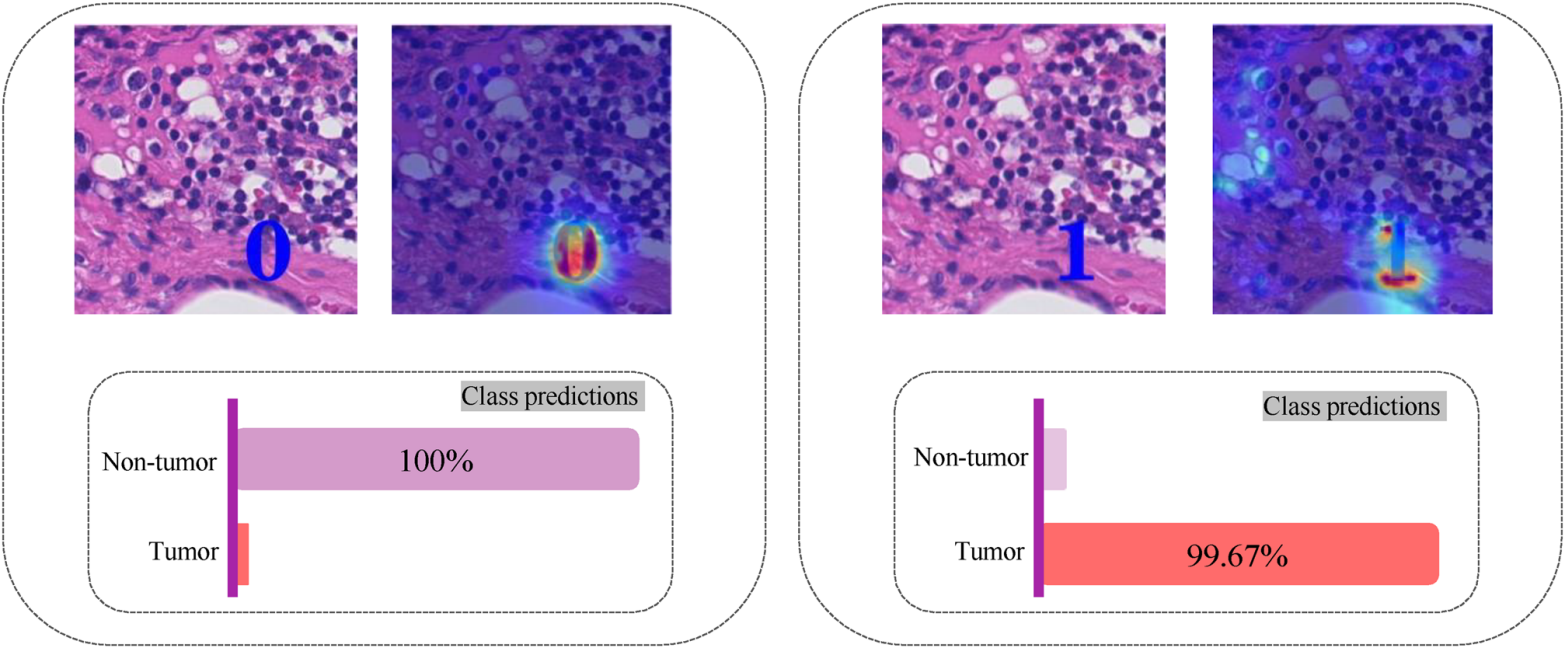
KimiaNet trained using *Scenario 3* when tested with a healthy (non-tumor) OOD patch with different class labels overlaid and their corresponding GradCAM heatmaps. (left) When true label (0: non-tumor) has been overlaid on the image. The network, by relying on the shortcut, classified the patch with confidence. (right) When the false label (1: tumor) has been overlaid. According to the class prediction of the network, the network has thoroughly paid attention to the overlaid digit and misclassified the image with its misleading shortcut.

GradCAM^53^ was used to provide some explainability such that providing heatmaps containing the salient areas relevant to the classification. According to Figs. 4 and 5, the GradCAM heatmaps show that *scenario 3* caused the deep network to focus on the overlaid label and failed to pay attention to the tissue morphology.

In other words, the extreme case of a shortcut which can be the overlaid label of the image takes precedence over any other content of the image in these cases. The deep network in these cases, similar to a digit recognizer, can only make a decision based on the digit overlaid on the image. When the overlaid class label is missed or misleading the deep network cannot provide satisfactory results.

We also tested the KimiaNet trained using *scenario 3* with images without class labels overlaid. The result was the deep network randomly generated class labels and similar to flipping a coin, the accuracy was ≈ 50%.

#### Scenario 1 and 2

We trained KimiaNet by holding out the hospital 3 images and using both *scenarios 1 and 2*.

**Figure 6:**
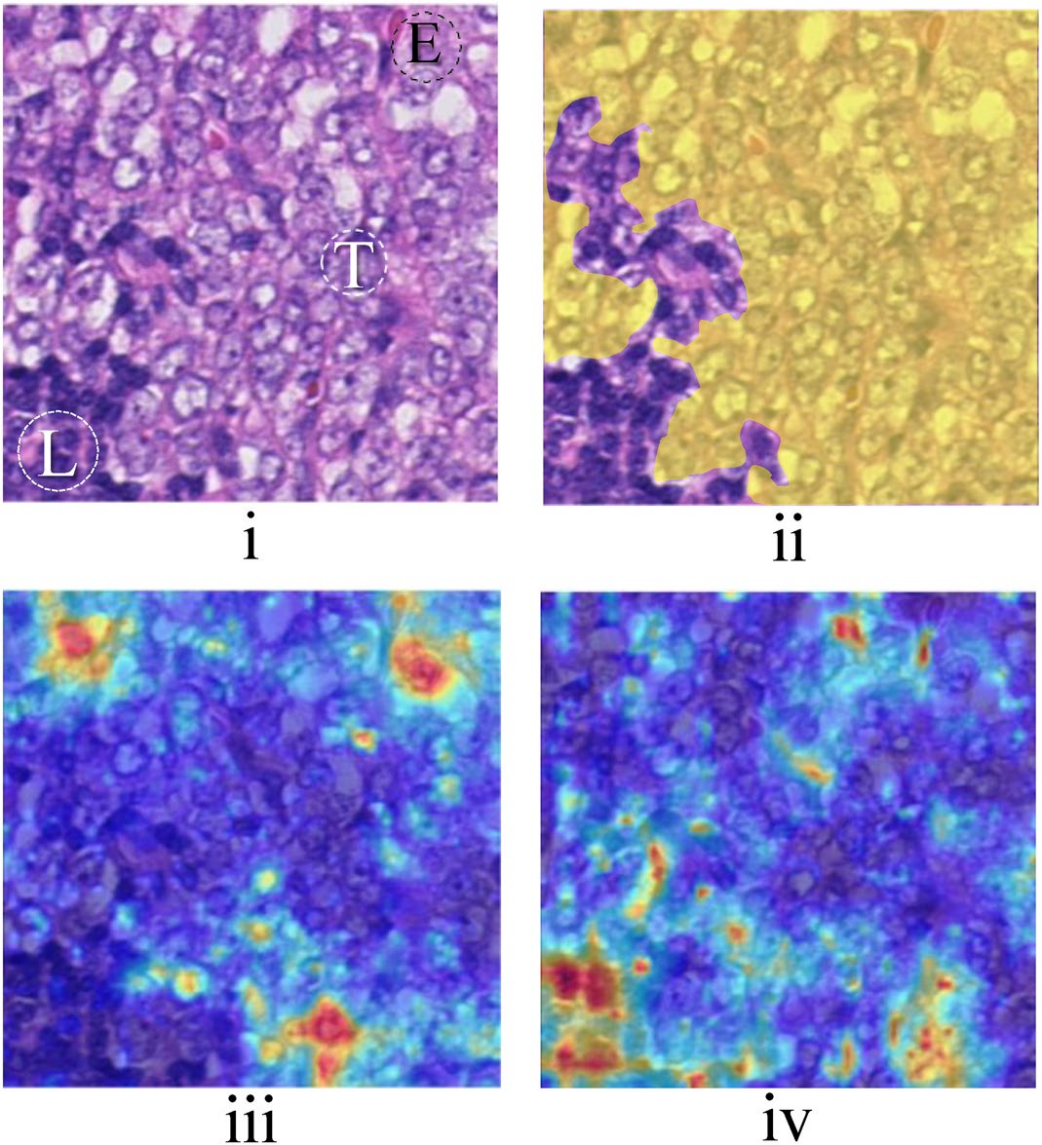
The result of training using *scenario 1* and *scenario 2*: (**i**) an OOD tumorous patch (from hospital 3) with different anatomical structures, Ⓣ: Tumor cells, Ⓛ: Lymphocyte, Ⓔ: Erythrocyte. (**ii**) Expert annotation for tumorous regions. (**iii**) GradCAM heatmap for the model trained using *scenario 2* which correctly classified the patch, (**iv**) GradCAM heatmap for the model trained using *scenario 1* which misclassified the patch as a healthy patch.

An OOD tumorous patch from hospital 3 with pathologist pixel-level annotation for the tumorous area is shown in Fig. 6i–ii. The model trained using *scenario 2*, correctly classified the image as tumorous and Fig. 6iii shows its salient tumorous area. While The model trained using *scenario 1* misclassified the patch as healthy, the explainability heatmap for salient healthy areas is shown in Fig. 6iv. Although some tumorous areas are missed in the explainability heatmap (Fig. 6iii), the activated areas correlate well with the expert annotation whereas lymphocyte areas have not been activated. In contrast, the model trained using *scenario 1* misclassified the patch as healthy; Fig. 6iv shows its heatmap for salient healthy regions. Although this heatmap is highly correlated with the healthy area (according to Fig. 6ii) but some tumorous regions erroneously have been activated as well. These regions may be attributed to shortcut opportunities that have been eliminated using the transformations of *scenario 2*.

**Figure 7:**
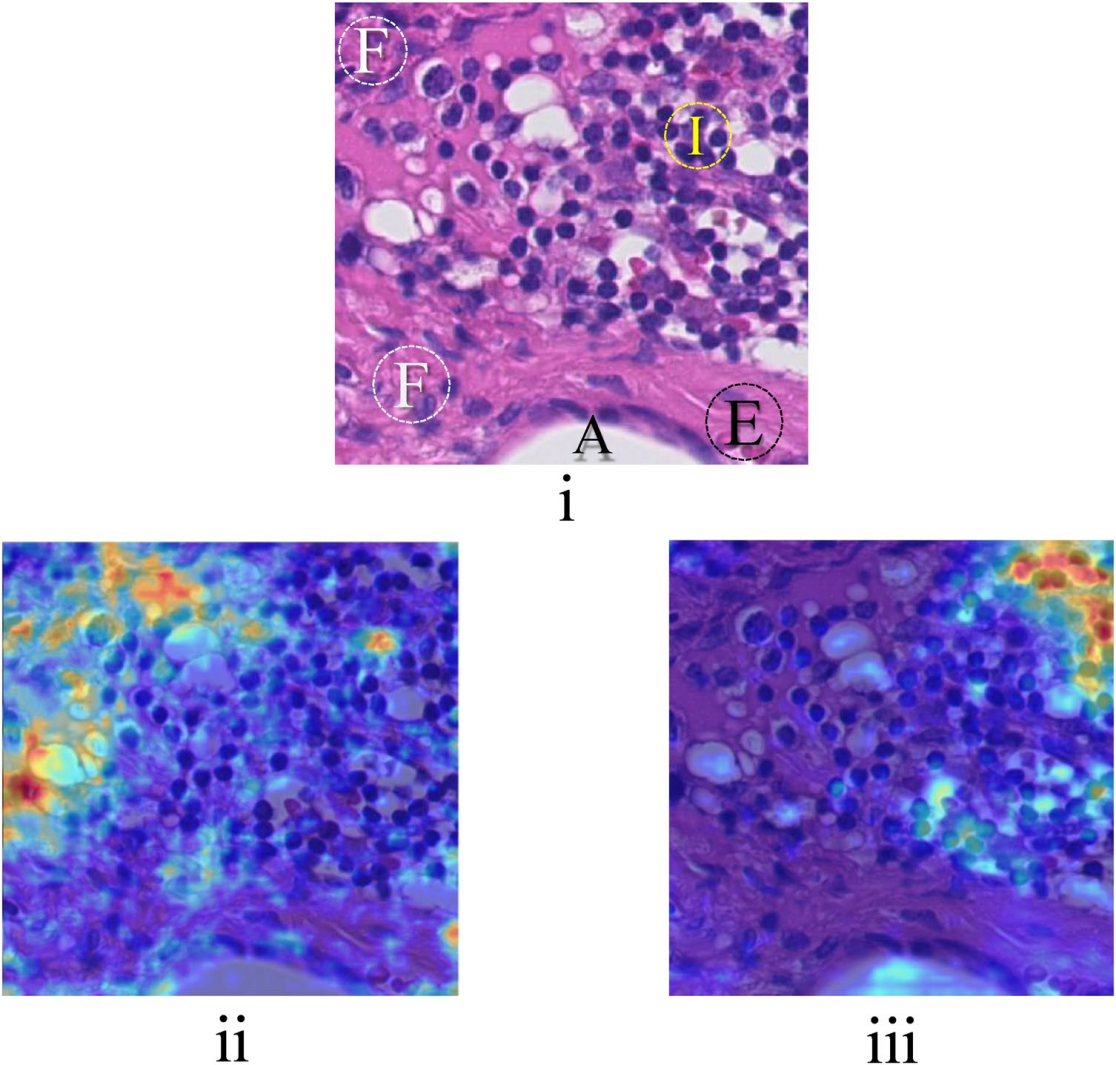
(**i**) an OOD healthy patch with different anatomical structures, Ⓘ: Immune cells, Ⓐ: Adipocyte, Ⓕ: Fibrous tissue, Ⓔ: Erythrocyte. (**ii**) GradCAM heatmap for the model trained using *scenario 1* which misclassified the patch as a healthy patch. (**iii**) GradCAM heatmap for the model trained using *scenario 2* which correctly classified the patch.

Figure 7i shows a patch containing healthy tissue. The trained network using *scenario 1*, erroneously classified this image as tumorous while the model trained by *scenario 2* correctly classified it as healthy. Figure 7ii and iii show heatmaps for salient tumorous and healthy areas for *scenario 1* and *scenario 2*, respectively. As it can be seen, the shortcut-trained model, or the model trained using *scenario 1*, has correlated fibrous tissues with the tumorous region. While in the model trained using *scenario 2* salient healthy areas are mostly immune cells and adipocytes. Thus, it can be observed that training using *scenario 2* defocused the deep network on non-semantic features (induced by spurious variations in stain colors or differences in morphology and tumor staging across hospitals/trial sites) rather than what we intend to, that is the semantics of tumorous or healthy patterns.

### Different pre-training: paying attention to different image aspects

#### Pre-training on a related task vs. ImageNet

While pre-training on natural images, such as vanilla, SSL, and SWSL pre-trained weights, has been dominant for many computer vision tasks, there is evidence to suggest that domain-specific pre-trained weights may be more effective for certain tasks^54,55^. Accordingly, it is likely that a pre-trained model on a comprehensive histopathology task, e.g., cancer subtyping on TCGA, would perform better than ImageNet pre-training for a histopathology downstream task, i.e., tumorous vs. non-tumorous breast tissues on CAMELYON. This is perhaps because histopathology images have unique characteristics, such as variation in cell structures and tissue patterns, that may not be well represented in ImageNet, which is a dataset of natural images. Through pre-training on TCGA, the model would have learned more relevant features and patterns for better performance on histopathology downstream tasks.

Moreover, KimiaNet has been trained on all the common cancer types from various hospitals such as Memorial Sloan Kettering Cancer Center (MSKCC) and National Cancer Institute Urologic Oncology Branch (NCI), as well as others. Through Empirical Risk Minimization (ERM) with the labels being cancer subtypes, the trained representations can be considered hospital-invariant to some extent. The variation among hospitals can indeed act as a form of data augmentation that can indirectly help improving the generalization of the KimiaNet. As a result, pre-training on TCGA can end up *overlooking* some irrelevant hospital-specific aspects of the images way better than pre-training on ImageNet. To support this hypothesis, heatmaps produced by XAI techniques were utilized in the following manner.

#### Heatmaps of the initial layers

**Figure 8:**
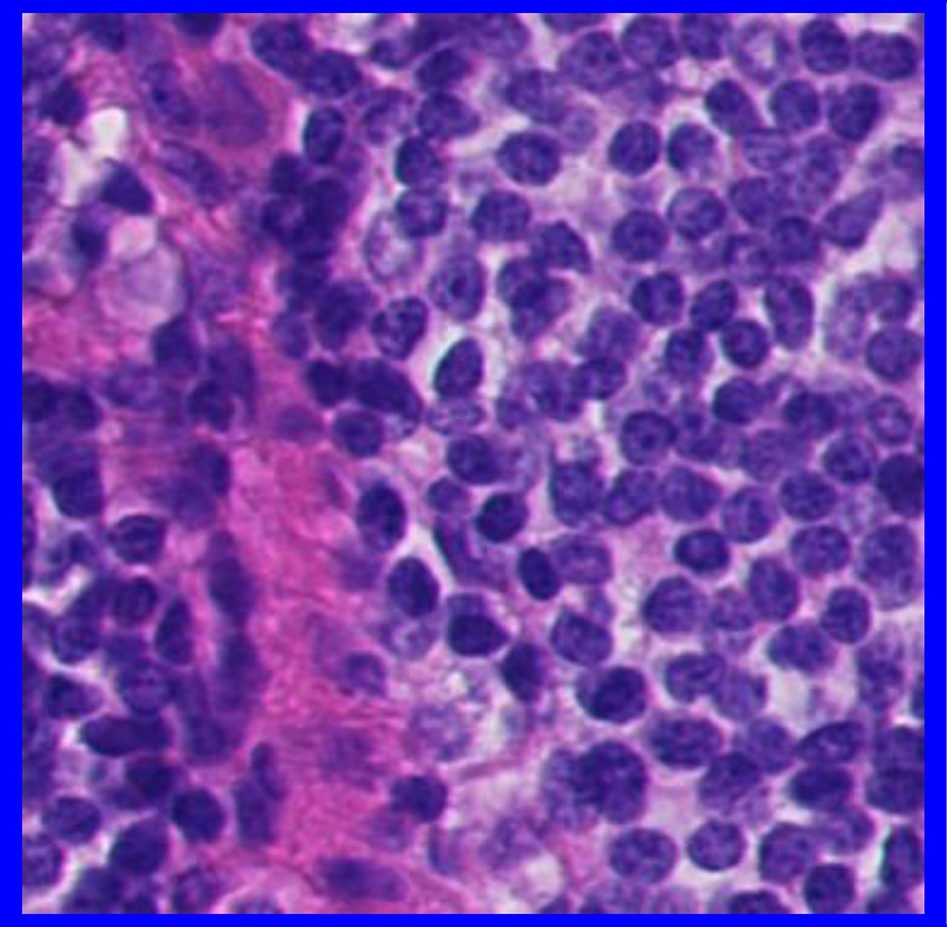
Sample non-tumorous patch at 20 × magnification from Hospital 3.

**Figure 9:**
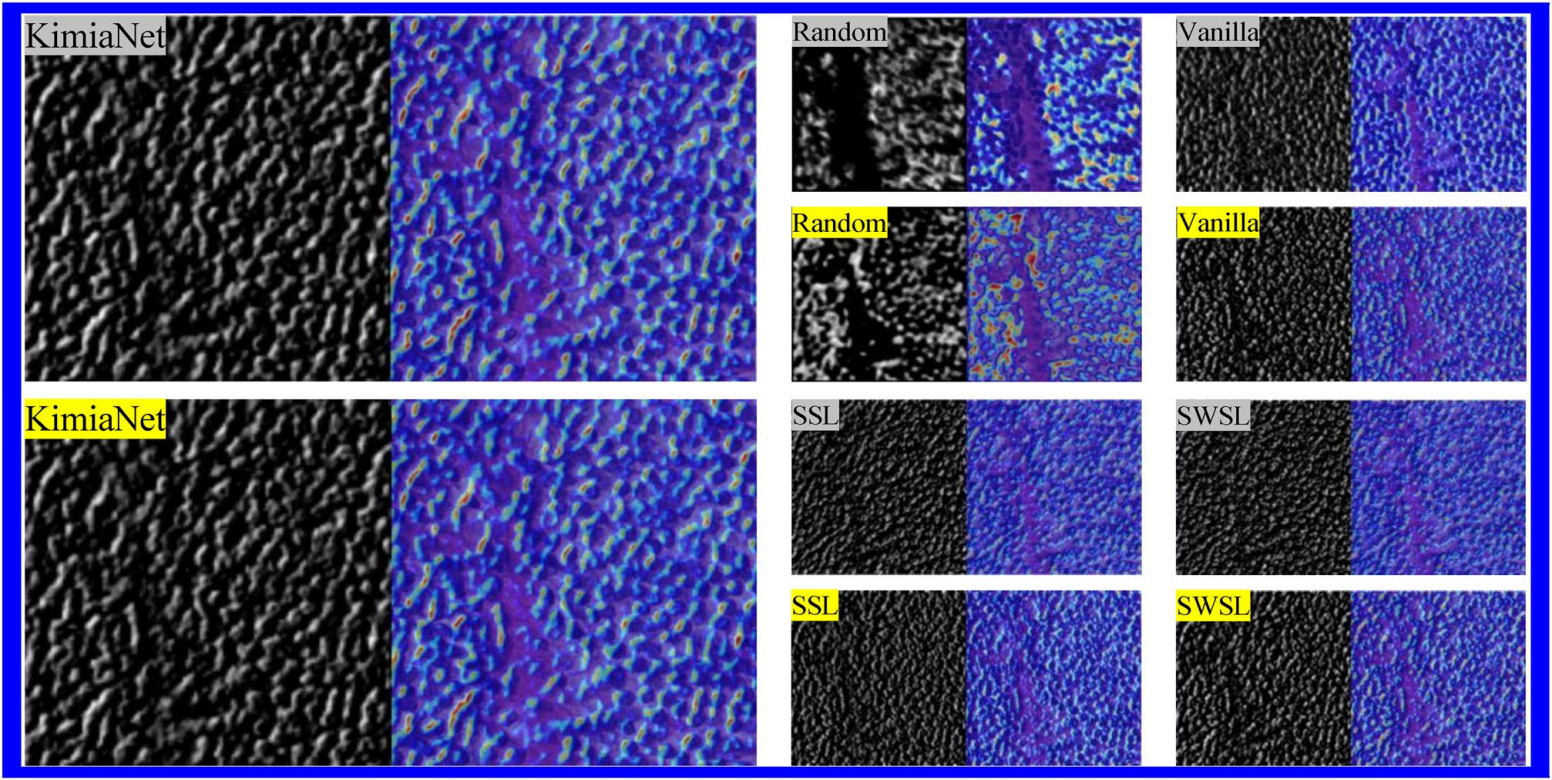
Activation maps of first layer weights: pre-trained weights (Gray-highlighted) and fine-tuning (Yellow-highlighted) using the same downstream task for each pre-training scenario.

The heatmaps generated by XAI techniques, especially GradCAM in this study, for the initial layers tend to highlight low-level features such as edges and corners in comparison to deeper layers which are more abstract and high-levels. These initial layers are usually left unchanged when fine-tuning a well-suited pre-trained model for the problem at hand, as they have already learned to detect “useful” features that are likely relevant to the new downstream task^56^. Accordingly, a crucial aspect of determining whether pre-trained weights are well-suited for downstream tasks is to assess whether fine-tuning induces significant changes to the initial layers. In this study, we investigated this issue by analyzing GradCAM heatmaps of the image shown in Fig. 8 for the first layer of each pre-trained model before and after fine-tuning (Fig. 9). Our results indicate that *the initial layer responses of KimiaNet remain consistent after fine-tuning* on an OOD healthy patch belonging to hospital 3, suggesting that the features captured by this pre-trained model are well-suited for the downstream task. However, for the other pre-trained models, changes in initial layer responses were observed, with the random weight model displaying the most dramatic changes. These findings suggest that careful consideration should be given to the choice of pre-trained weights for downstream tasks.

## Conclusions

Although a fixed-policy diversification of images, similar to *scenario 2* in this study, may lead to OOD generalization improvement, that is not necessarily the case. We showed that in some cases the data diversification, counter-intuitively, leads to poor OOD and in-distribution performance due to complicating the training of the deep networks. Hence, it is not always possible to a priori assume a policy that fits all scenarios unless the target test data and its distribution are available/known. A good example is the learnable augmentation policies^57^ using Cycle-Generative Adversarial Networks (Cycle-GANs)^58^ which is utilized for adapting the target data to source data for improving the OOD generalization. However, in this study, we assumed that there is no access to the target data during the training.

Although there are some works claiming that pre-training is not sufficiently effective, this paper showed that the use of pre-training in computer vision should not be dismissed. We have demonstrated that the newly released pre-trained vision models (SWSL, and SSL) do improve performance in many scenarios as other works have already shown that^49^. Additionally, we showed that KimiaNet which is a histopathology-tailored pre-trained model can outperform pre-trained models tailored toward natural images by far when the distribution shift is significant and the domain of study is histopathology.

We utilized XAI techniques to provide explanations and interpretations for certain conclusions. We presented empirical evidence that data diversification could enhance OOD performance by eliminating shortcuts, and investigated how the suitability of various pre-trained models affects the activation maps of the initial layers in deep networks.

Although some of these conclusions may be obvious, this paper presented a thorough examination of various histopathology trial site repositories, pre-trained models, and image transformations. Moreover, the paper could serve as a reference for practitioners who are not acquainted with the prevailing ideas in the field. It seems it is a common practice among the computational pathology community is to utilize ImageNet pre-trained models for their histopathology downstream tasks.

### Limitations

Although our study extensively examined the performance of various pre-trained models on OOD test data in histopathology repositories, it is important to acknowledge its limitations. Firstly, the study only applied ERM on different pre-trained models and did not explore other approaches such as domain adaptation and domain generalization that may offer better generalization on OOD data. Secondly, while XAI techniques were employed for interpreting the results, the explanations generated were not thoroughly analyzed. A more comprehensive investigation of these explanations could provide deeper insights into the causes of the distribution shift in histopathology domains.

Furthermore, our study only considered a limited set of pre-trained models, including vanilla ImageNet, SSL, SWSL, and KimiaNet pre-trained models. There are many other pre-trained models designed for different tasks. Therefore, the results may not be generalized to all pre-trained models.

## Data Availability

The dataset CAMELYON17 analysed during the current study is available in the Grand Challenge repository: https://camelyon17.grand-challenge.org/.
